# Supplementary figures and images for: The Osteoblast Transcriptome in Developing Zebrafish Reveals Key Roles for Extracellular Matrix Proteins Col10a1a and Fbln1 in Skeletal Development and Homeostasis
Source: Biomolecules. 2024 Jan 23;14(2):139. doi: 10.3390/biom14020139 (PMC10886564; doi:10.3390/biom14020139)

## Slide 1
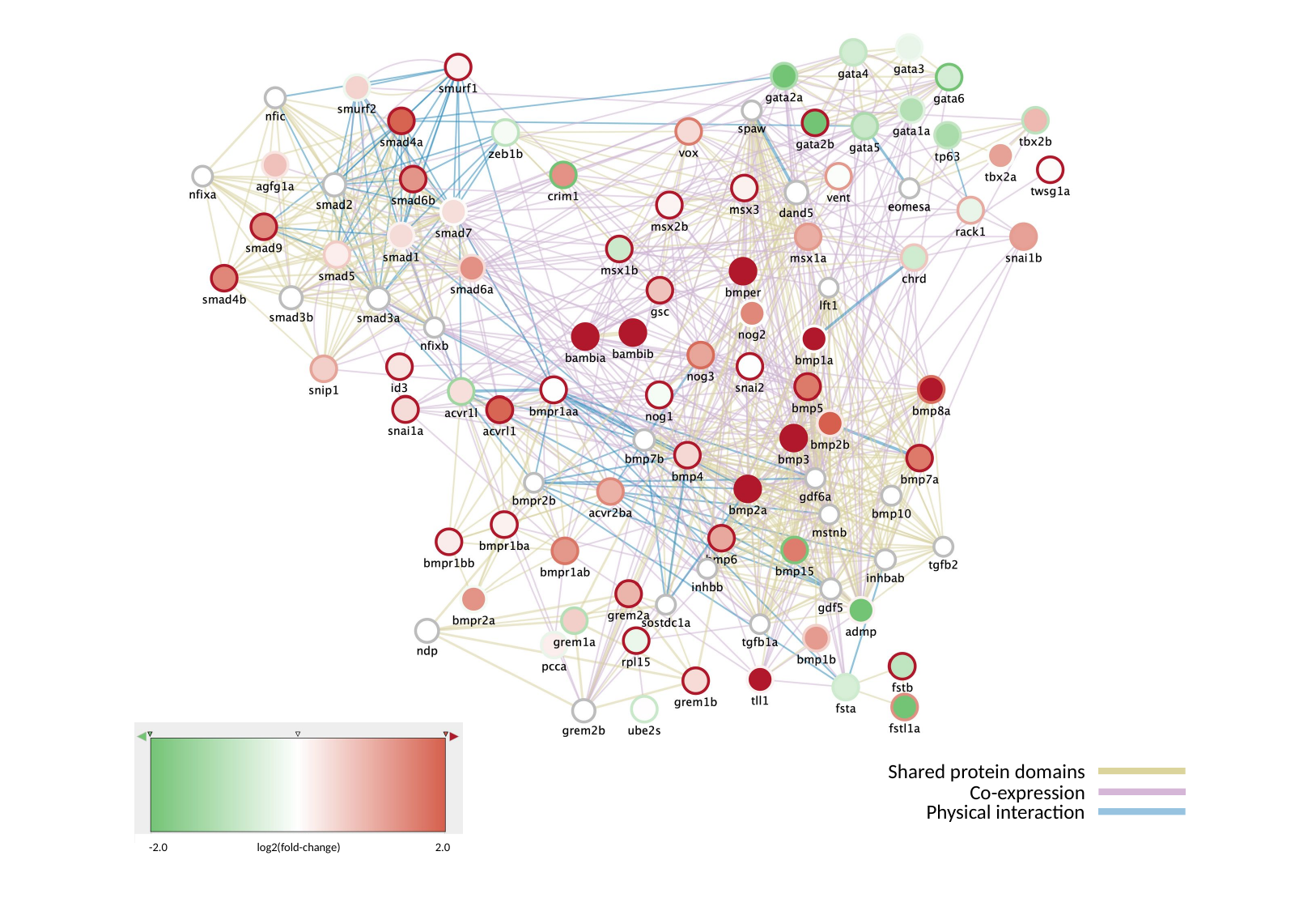

log2(fold-change)
-2.0
2.0
Shared protein domains
Co-expression
Physical interaction

Supplement: Supplementary file 1 [file biomolecules-14-00139-s001.zip › Figure S1 osteoblast BMP .pptx]
